# The external Ballard examination does not accurately assess the gestational age of infants born at home in a rural community of The Gambia

**DOI:** 10.1179/146532810X12786388978526

**Published:** 2010-09

**Authors:** R. A. M. Taylor, F. C. Denison, S. Beyai, S. Owens

**Affiliations:** * MRC Keneba Field Station, The Gambia; † Centre for Reproductive Biology, Queen's Medical Research Institute, University of Edinburgh, Edinburgh; ‡ The Liverpool School of Tropical Medicine, Liverpool, UK

## Abstract

**Background:**

Accurate assessment of gestational age at birth is critical to the identification of neonates at high risk. In resource‐poor settings, postnatal techniques are commonly used but may be difficult to apply and have not been well validated against ultrasound in community studies. The aim of this study was to evaluate postnatal assessment of gestational age in rural Africa using the external criteria of the Ballard examination against 1st/early 2nd‐trimester ultrasound and date of last menstrual period.

**Method:**

In a sample of women from Kiang West, The Gambia (*n* = 80), the precision of gestational age estimates using the external Ballard examination was compared with those derived from 1st and early 2nd‐trimester ultrasound examination and date of last menstrual period.

**Results:**

The incidence of preterm delivery was low at 2.5%. The external Ballard examination tended to underestimate gestational age by a mean (SD) of 15.6 (10.9) days compared with that derived from ultrasound and to underestimate by 15.4 (23.1) days compared with that derived from date of last menstrual period. The differences between the methods varied with gestation.

**Conclusion:**

In this rural, community‐born population of infants, postnatal assessment of gestational age by external Ballard examination performed poorly compared with ultrasound and last menstrual period. No reliable gestational age could be derived from its estimate and it failed to detect a significant proportion of high‐risk infants. The development of an accurate but simple method of postnatally assessing gestational age specifically for use by health workers in rural Africa is required.

## Introduction

Many of the four million newborns who die each year, mostly in developing countries, are born prematurely, growth‐restricted or both.^1^ Deaths which occur during the perinatal period in poor rural communities are particularly difficult to prevent and are a direct challenge to the achievement of the 4th Millennium Development Goal (MDG).^2^ Knowledge of the gestational age (GA) of neonates at birth is important in order to identify and categorise those born at high risk of poor perinatal outcome who might benefit from closer clinical support, particularly in such settings.

Sonographic determination of the estimated date of delivery (EDD), based on fetal growth parameters measured during a 1st or early 2nd‐trimester ultrasound scan (USS), is considered to be the ‘gold standard’ method of pregnancy dating but is seldom available in rural sub‐Saharan Africa. Clinical dating based on the last menstrual period (LMP) or measurement of fundal height is frequently inaccurate.^3^ Instead, ordinal observations recorded on postnatal examination of the newborn baby are often used to calculate GA according to standardised scoring charts. The best known of these techniques is the Dubowitz examination which scores infants on 11 external and 10 neurological criteria.^4^ Although widely used, Dubowitz is a relatively complex technique and considerable clinical experience and training are required to detect slight changes in neonatal posture and muscle tone. It has been observed that Dubowitz and other clinical methods which score both neurological and external criteria underestimate GA in small‐for‐gestational‐age (SGA) and term infants.^5^ This might reflect the difficulty of performing the neurological component in the 1st few days of life when birth shock or birth‐related trauma (e.g. breech delivery) can alter tone and reflexes, thereby biasing the neurological component. Such difficulties may limit the applicability of Dubowitz in the field, especially for non‐specialists working in resource‐poor settings.

An alternative, simplified postnatal scoring system was proposed by Ballard and has been further adapted.^6^ A single, hospital‐based Malawian study found that nurse‐led GA assessments derived from LMP, uterine fundal height examination and Dubowitz score compared favourably with those obtained from a modified Ballard examination in which only the six external criteria were scored, termed the ‘external Ballard examination’ (EBE).^7^ However, we undertook a small pilot study in 45 infants born across a limited range of gestational ages in rural Gambia and found that the score derived from EBE performed by a newly‐trained medical student correlated very poorly with that derived from a Dubowitz examination performed by an experienced midwife and with that derived from USS.^8^ To date, there are no published studies comparing EBE with the gold standard of 1st or early 2nd‐trimester USS in a resource‐poor setting.

A simple postnatal examination to identify neonates at risk of fetal growth restriction or prematurity which is applicable at community level rather than in hospital would be of great benefit to health workers required to make clinical decisions about referral for limited but more specialised paediatric care.

The aim of this study was to evaluate the utility of EBE performed by an experienced midwife in a rural African setting. The specific objective was to compare postnatal assessments of gestational age by EBE with those derived prenatally by USS.

## Methods

### Location

The study was conducted at the Medical Research Council's (MRC) station in Keneba, Kiang West, an isolated district of Lower River Division in The Gambia. Kiang West is a holo‐endemic malarial zone inhabited predominantly by ethnic Mandinka who typically exist as subsistence cultivators. Antenatal health care is provided by government nurse‐trekking teams in collaboration with the MRC.

### Study design

This method‐comparison study was nested within a randomised, controlled trial investigating the effect of periconceptual multi‐micronutrient supplementation on placental function in rural Gambian women (Periconceptional Multiple Micronutrient Supplementation Trial, PMMST) (ISRCTN 13687662); all data were collected as part of the existing protocol for that study. Postnatal GA assessments were derived from EBE performed by an experienced community midwife (SB) who was previously familiar with the Dubowitz technique and who had been trained in EBE. These were compared with GA assessments derived from 1st/early 2nd‐trimester ultrasound measurements performed by a trained clinician (SO), and from LMP. The midwife was blinded to the results derived from USS and LMP.

All liveborn, singleton infants born between 1 May and 30 November 2007 with sonographically determined EDD and without major congenital anomalies were eligible for inclusion. Recruitment was opportunistic and not based on prior calculation of sample size.

Dating USS was performed at antenatal booking visits when LMP was also recorded. All neonates were seen and assessed using EBE within 72 hours of birth.

### Estimates of gestational age

EBE scored neonates on the six observational, external criteria described in the full Ballard examination: skin appearance, presence of lanugo hair, plantar creases, breast tissue, ear formation, and external genitalia formation. The range of possible scores was ‐8 to 25. EBE scores were then doubled to account for the absence of neurological assessment, and GA extrapolated from the standardised Ballard scoring chart.^6^

Training in EBE was provided by a paediatrician (SO). Data collection for the study did not begin until the EBE scores attributed by SB and SO on ten consecutive deliveries had achieved good correlation (R^2^ = 0.7).

GA by USS was derived from crown–rump length (CRL) measurements at less than 14 weeks gestation, and biparietal diameter (BPD) measurements at 14–24 weeks gestation using charts validated in African populations.^10^ Women who presented over 24 weeks gestation were not dated using USS as measurements beyond this point were considered unreliable. Such women had their pregnancies dated using LMP.

GA by LMP was calculated from the dates given by pregnant women at booking. Neonates were classified as term (⩾37 weeks) or preterm (<37 weeks). SGA infants were defined as those with a birthweight below the 10th centile for gestation based on standardised charts.^11^

After GA had been assessed, infants' weights were measured to the nearest 10 g using a spring balance.

### Statistical analysis

Data analysis was performed using Microsoft Excel and Graph Pad Prism (Graphpad, USA). Data were entered using Microsoft Access database software. Bland–Altman plots were used to assess agreement of gestational age estimates.^12^ The mean of the bias±1.96 standard deviations gives the 95% limits of agreement when the differences are normally distributed. Data are presented as means and standard deviations (SD). Statistical significance was taken as *p*<0.05.

### Ethics

The study was approved by the MRC Scientific Coordinating Committee and by the Gambian Government Ethics Committee. Informed consent was obtained from all mothers at recruitment.

## Results

### Demographics

**FIG. 1:**
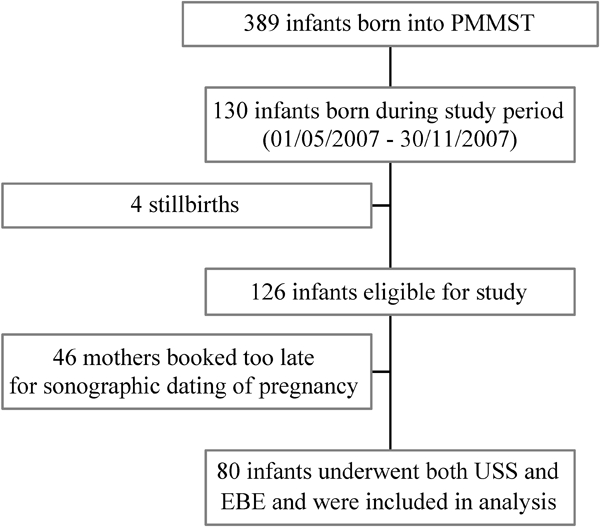
Flowchart illustrating recruitment for the study.

A flowchart of subject recruitment is shown in Fig. 1. During the study period (1 May to 30 November 2007), 80 neonates were recruited (42 males and 38 females); mean (SD) birthweight was 2939 (429) g. LMP was available for only 76 (95%) of the 80 women. Mean (SD) GA at booking was 104 (33.8) days. EBE was performed within 72 hours on all 80 neonates.

### Estimates of gestational age

Table 1 shows the estimates of gestational age. EBE classified a greater number of neonates as preterm (20/80, 25%) than LMP (14/76, 18.4%) or USS (2/80, 2.5%).

**TABLE 1:** Estimates of gestational age by different methods

Method	No.	Mean GA (d)	SD	Range (d)
USS	80	281.3	11.1	247–313
LMP	76	280.7	23.1	214–347
EBE	80	265.7	7.5	252–286

### Comparison between methods

**FIG. 2:**
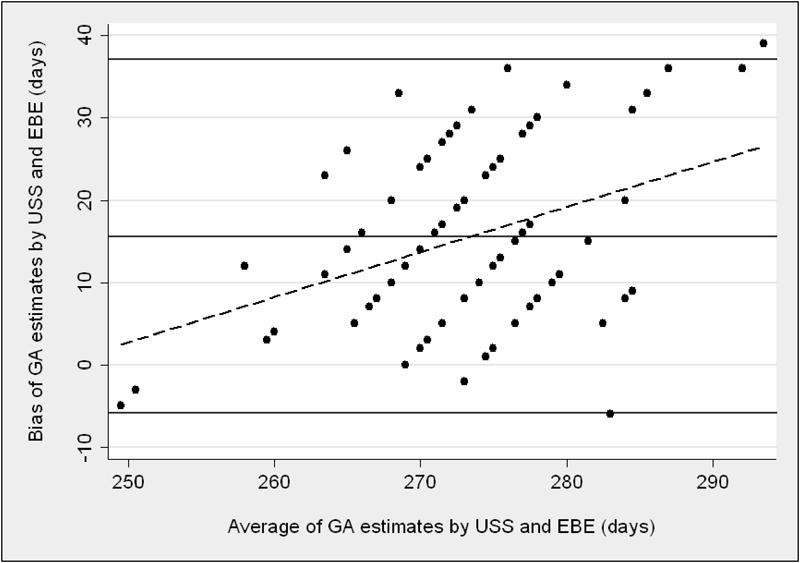
Agreement between GA derived from USS and EBE. The solid horizontal lines represent the mean bias and 95% limits of agreement between the methods. The dashed line represents the linear regression line between bias and average gestational age of the methods compared.

**FIG. 3:**
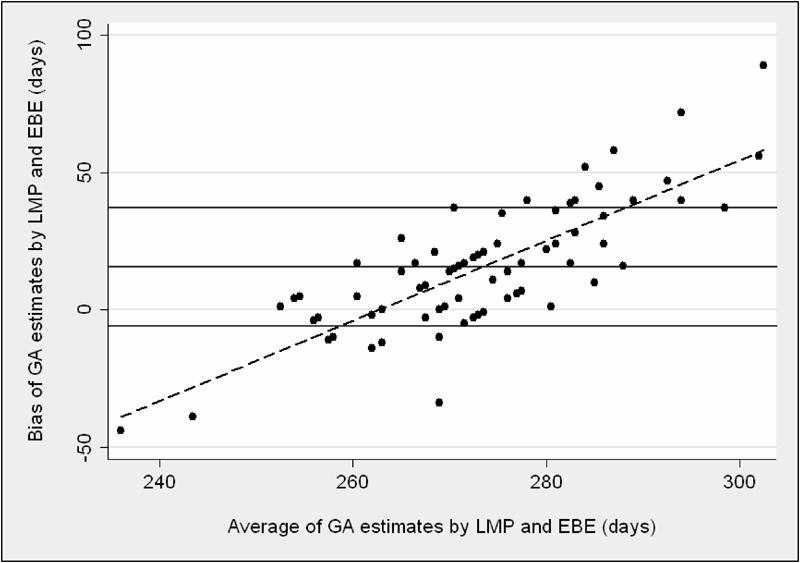
Agreement between GA derived from LMP and EBE. The solid horizontal lines represent the mean bias and 95% limits of agreement between the methods. The dashed line represents the linear regression line between bias and average gestational age of the methods compared.

Figs 2 and 3 demonstrate the bias of the different gestational age estimates. Fig. 2 shows that USS‐generated assessments of GA were a mean (SD) 15.6 (10.9) days longer than the EBE estimates. Limits of agreement were wide (−5.9 to +37.1 days). Linear regression of the bias gave a correlation co‐efficient of 0.40.

Fig. 3 shows that LMP‐estimated GA exceeded EBE estimates by a mean (SD) 15.4 (23.1) days. Limits of agreement were again wide (−30 to +61 days). Linear regression gave a correlation co‐efficient of 0.81 to the bias.

### Growth restriction

The distribution of SGA diagnoses by EBE and USS is illustrated in Table 2. USS identified 28/80 (35.0%) neonates as SGA while EBE identified 11/80 (13.8%) infants as SGA. The sensitivity of EBE for detecting SGA infants was 35.7%. The specificity of EBE for detecting SGA infants was 98.1%.

**TABLE 2:** Numbers of SGA infants as defined by EBE and USS

	SGA on USS	Not SGA on USS	Total
SGA on EBE	10	1	11
Not SGA on EBE	18	51	69
Total	28	52	80

Linear regression showed that the bias between EBE and USS assessments of GA was unrelated to birthweight. (F = 0.00, *p* = 0.966, R^2^ = −0.013).

## Discussion

The aim of this study was to evaluate the applicability of EBE used in a community setting (as opposed to hospital) in a rural West African population. We have demonstrated that GA assessments derived from EBE performed by a midwife compared poorly with those derived from the ‘gold standard’ of antenatal USS. We therefore conclude that EBE is not a reliable method of assessing GA in this resource‐poor community setting and other methods ought to be explored.

To estimate the level of agreement between two GA assessments, Bland–Altman analysis was used to plot the difference between the methods against the average in order to give the bias. Correlation statistics were not used because they measure the strength of a linear association and not the level of agreement between two methods.^12^

Using this technique, the mean bias between USS and EBE was 15.6 days with unacceptably wide limits of agreement. However, bias between the two methods increased significantly as GA increased, such that there was progressive underestimation of GA by EBE at 40 weeks gestation and beyond. Conversely, bias tended to decrease before 40 weeks. This finding is in keeping with the fact that EBE correctly identified the two infants who were born preterm but also incorrectly identified a further 18 term infants as preterm.

EBE likewise underestimated GA derived from LMP by a mean 15.4 days and the bias increased as GA increased. This confirmed a previous study by Verhoeff *et al.* who found that when GA exceeded 270 days, LMP overestimated GA by a mean 12.7 days (literate women) and 18.8 days (illiterate women).^7^ LMP is generally thought to be a poor indicator of GA in communities where female illiteracy is common.^3^ The fact that 95% of women in this study knew their LMP is significant and is much higher than in the study by Verhoeff *et al.* who obtained LMP data in fewer than 15% of cases.^7^ The finding may reflect the influence of midwife‐led antenatal teams which have been active in the region over the last 20 years, supported by the clinical staff and funds of MRC Keneba.^13^ Recently published qualitative data suggest that women in Kiang West do detect signs of their pregnancies early but fail to disclose their status for social reasons, with obvious clinical implications.^14^

The incidence of SGA defined by USS was high (35%) and confirms the previously described peak incidence of 31% in November, defined using the Dubowitz scoring.^15^ The high prevalence of SGA reflects the impact on fetal growth of the annual ‘hungry’ season, the severity of which peaks in October when there is a marked deterioration in maternal nutritional status owing to food scarcity, compounded by increased intensity of agricultural labour and seasonal epidemics of infectious diseases.^15^ EBE was of limited use in detecting SGA neonates, identifying only 35% of those affected, although with high specificity (98%). An unacceptable number of growth‐restricted infants would be missed if GA were derived solely from EBE in our population.

One of the significant problems with EBE is that one incorrect score shifts the calculated GA by 5.6 days as opposed to 1.8 days with Dubowitz, for example.^4^ Assessment of variation in skin colour in the African newborn is problematic, especially when performed in unlit homes, and this is a major limitation. Underestimating GA by over 2 weeks or more may lead to neonates being incorrectly categorised as ‘at‐risk’, with unnecessary clinical intervention and subsequent misallocation of limited resources.

Infants in this study were born within a narrow range of gestational ages (as defined by USS ultrasound, 247–313 days) with most infants born at or around term and there was a low incidence of prematurity (2.4%). Our population therefore contrasts with that described by Verhoeff *et al.* which consisted of infants delivered in a large urban hospital and which spanned a maximum GA range of 135–349 days, based on fundal height measurements.^7^ It is possible that a larger, more diverse study population which included more premature infants would have generated overall better correlation, smaller bias and narrower limits of agreement between the methods of GA assessment in our study. However, to be considered effective in a community setting, where most infants who survive long enough to undergo clinical review are born at or around term, such techniques must retain precision within the same range.

The main weakness in this study is the use of a single operator to assess GA using EBE. It could be argued that the poor precision of the estimates was primarily a function of the examiner rather than the examination. However, the midwife chosen to carry out the measurements was experienced in assessing newborn infants and there was good correlation between the EBE scores calculated by himself and the paediatrician for ten infants assessed before the study commenced. The midwife in question might be considered typical of those working in rural community settings in many parts of West Africa. The findings in the current study were also supported by those in our previous analysis in which EBE compared poorly with Dubowitz.^8^

Postnatal GA assessments were the focus of a great deal of attention in the 1970s, pre‐dating the advent of accurate sonographic dating. As developed countries move away from the use of such methods, their focus in the literature has dwindled; however, the need for accurate postnatal assessments in developing countries, particularly in rural settings, has not changed. Further validation of their applicability in the field is essential. While some investigators have been impressed by the correlation between Dubowitz and LMP^16^ in African infants, few studies have measured the actual level of agreement between prenatal and postnatal techniques, and to our knowledge none has done so against the gold standard of antenatal sonography. Others have been critical of Dubowitz because of its complexity and a tendency to underestimate GA in growth‐restricted infants.^5^

By assessing the use of EBE in the community, we have tested the applicability of a simplified method of postnatal GA assessment in a setting where the results are most relevant. Whilst we are able to comment only on its applicability across a narrow range of GA, EBE does not appear to be a suitable method for our population. If our findings are repeated, we would propose the development of a simplified postnatal scoring system, adapted for the African newborn, specifically for use by community health workers.
